# Downregulation of SMOC2 expression in papillary thyroid carcinoma and its prognostic significance

**DOI:** 10.1038/s41598-020-61828-z

**Published:** 2020-03-17

**Authors:** Hye Sung Kim, Jae Hyuck Choi, Jae Young Lee, JiHoon Kang, Jae Kyung Myung, Woo Ho Kim, Bo Gun Jang

**Affiliations:** 1 Department of Pathology, Jeju National University School of Medicine and Jeju National University Hospital, Jeju, South Korea; 2 Department of Surgery, Jeju National University School of Medicine and Jeju National University Hospital, Jeju, South Korea; 3 Laboratory of Radiation Exposure & Therapeutics, National Radiation Emergency Medical Center, South Korea Institute of Radiological & Medical Sciences, Seoul, South Korea; 4 Department of Pathology, Korea Cancer Center Hospital, Seoul, South Korea; 5 Department of Pathology, Seoul National University College of Medicine, Seoul, South Korea

**Keywords:** Tumour biomarkers, Thyroid diseases

## Abstract

Secreted Protein Acidic and Rich in Cysteine (SPARC)-related modular calcium-binding protein-2 (SMOC2), a secreted matricellular protein, is reported to be involved in various processes related to cancer progression such as regulating the cell cycle, angiogenesis, and invasion. However, its expression and prognostic significance in papillary thyroid carcinomas (PTCs) remains unknown. Using immunohistochemistry, we evaluated the expression profile of SMOC2 and its prognostic value in a large cohort of PTCs. Real time-PCR analysis with fresh-frozen tissues showed that SMOC2 mRNA expression in PTCs was substantially lower than the expression in matched non-cancerous thyroid tissues, consistent with the results from thyroid cancer cell lines. Immunohistochemical analysis demonstrated that SMOC2 was normally present in thyroid follicular epithelial cells and the expression level was maintained in nodular hyperplasia. However, SMOC2 expression was significantly lower in lymphocytic thyroiditis and follicular tumors including follicular adenomas and carcinomas. In particular, 38% of PTCs exhibited a complete loss of SMOC2 expression, which was associated with the presence of *BRAF* (V600E) mutation. Moreover, SMOC2 further declined during lymph node metastasis in PTCs. DNA methylation chip analysis revealed one hypermethylated CpG site in the promoter region of SMOC2 gene, suggesting an epigenetic regulation of SMOC2 in PTCs. Remarkably SMOC2 positivity was associated with improved recurrence-free survival along with female sex, tumor size, and the N stage. However, SMOC2 was not identified as an independent prognostic marker in multivariate analyses. Taken together, SMOC2 expression is significantly down-regulated in PTCs and SMOC2 positivity is closely associated with better clinical outcomes, suggesting that SMOC2 can be a prognostic marker in PTC patients.

## Introduction

Thyroid cancer is the most common endocrine malignancy and its incidence is steadily rising worldwide, with the most common histology being papillary thyroid carcinoma (PTC). Common mutations found in PTCs are point mutations of *BRAF* and *RAS* genes as well as *RET*/*PTC* and *PAX8*/*PPARγ* chromosomal rearrangements, and these genetic alterations have been demonstrated to be diagnostic and prognostic markers^1^. Despite its indolent biologic behavior and excellent prognosis, PTC occasionally progresses to less differentiated and more aggressive subtypes. Moreover, as a significant number of PTC patients develop lymph node metastasis and locoregional recurrences, this group of patients need to be identified for more aggressive treatments to mitigate the progression of PTC.

To identify a molecular marker that can be useful for the prognostication of PTC patients, here we examined the expression of Secreted Protein Acidic and Rich in Cysteine (SPARC)-related modular calcium-binding protein-2 (SMOC2) in a variety of thyroid pathologies. Interestingly, SMOC2 was identified as a risk locus for autoimmune thyroid disease^2^ and SMOC2 single nucleotide polymorphism showed a marginally significant association in female autoimmune thyroid disease patients^3^. These results also suggest a possible implication of SMOC2 in thyroid tumors. SMOC2 was isolated as a matricellular protein that contains two thyroglobulin type-I domains, two EF-hand calcium-binding domains, and a follistatin-like domain^4^. SMOC2 belongs to the SPARC family and is widely expressed in many tissues^4^. SPARC regulates cell interaction with the extracellular environment during development and disease as well as in response to injury^5^. These biological effects are derived from interactions between growth factors, integrins, and extracellular matrix proteins^5^. Likewise, SMOC2 has been reported to be involved in a variety of cellular functions including cell adhesion and migration^6^, angiogenesis^7^, fibrosis^8^, and cell proliferation^9^.

However, there are only a few studies on the functional or prognostic significance of SMOC2 in human cancers. For instance, SMOC2 elevation was suggested to be necessary for L1-mediated induction of more invasive colorectal cancers^10^, and SMOC2 down-regulation greatly reduced the ability of Ran mutation to stimulate cell growth in the breast cancer line, SKBR3^11^. In hepatocellular carcinoma (HCC), Huang *et al*. showed that SMOC2 overexpression attenuated the tumorigenicity and was associated with better prognosis^12^ whereas Su *et al*. reported that overexpression of SMOC2 promoted HCC cell proliferation and cell cycle progression^13^. Most recently, it was demonstrated that SMOC2 expression in endometrial carcinoma was closely associated with the cancer stem cell (CSC) markers, CE133 and CD44, and the silencing of SMOC2 enhanced chemosensitivity^14^. As there has been no study on SMOC2 expression in thyroid cancers, here we aimed to investigate the expression profile of SMOC2 in various thyroid diseases including a large cohort of PTCs and to analyze the prognostic impact of SMOC2 as well as its correlation with clinicopathological features.

## Results

### SMOC2 expression in papillary thyroid carcinomas and normal thyroid tissues

**Figure 1:**
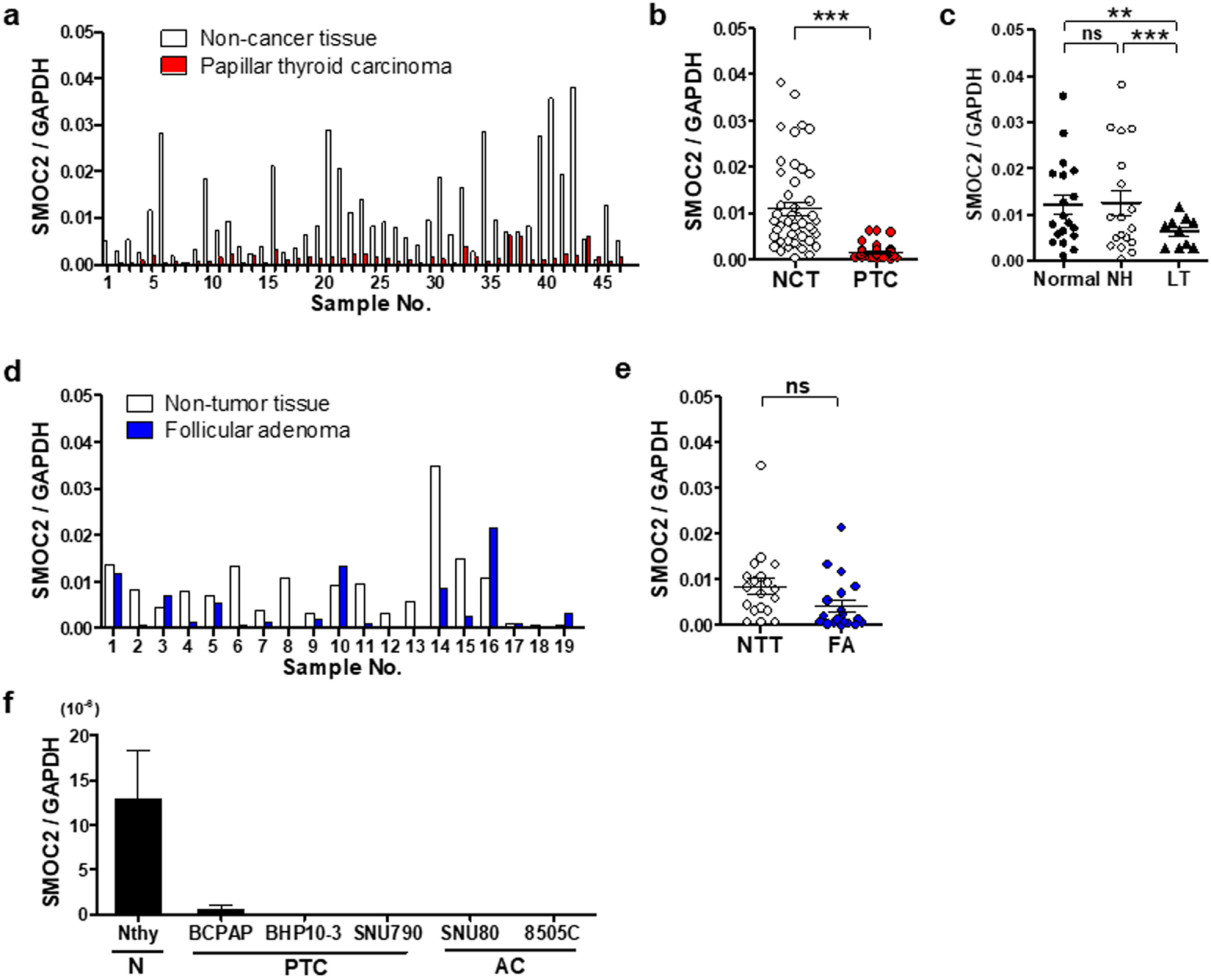
SMOC2 mRNA expression in papillary thyroid carcinomas (PTCs) and follicular adenomas (FAs). (**a**) Real-time PCR analysis was performed to examine the expression of SMOC2 with 47 pairs of PTC and corresponding non-cancer thyroid tissues. (**b**) The mean SMOC2 level in PTC and non-cancer tissues. (**c**) SMOC2 expression in normal thyroid, nodular hyperplasia, and lymphocytic thyroiditis. (**d**,**e**) SMOC2 mRNA level in FA and matched non-tumor tissues. (**f**) SMOC2 expression in normal thyroid and various thyroid cancer lines. NH, nodular hyperplasia; LT, lymphocytic thyroiditis; N, normal; AC, anaplastic carcinoma. Nthy, Nthy-ori 3-1; ns, not significant. **P < 0.01, ***P < 0.001.

To compare the expression of SMOC2 between PTCs and normal thyroid tissues, we collected a series of 47 pairs of fresh-frozen PTC samples and matched normal thyroid tissues. SMOC2 mRNA expression in PTC was lower than the expression in the matched normal tissue in the majority of samples (45 out of 47 cases, 94%) (Fig. 1a). The mean SMOC2 level was also much lower in the PTC samples than in adjacent non-cancerous tissues (*P* < 0.001) (Fig. 1b). Interestingly, when looking into 47 non-cancerous tissues, SMOC2 mRNA expression was significantly lower in lymphocytic thyroiditis than in nodular hyperplasia or normal thyroid tissues (Fig. 1c). SMOC2 expression was down-regulated in most FA cases (14 out of 19 cases, 74%) (Fig. 1d), and the mean SMOC2 expression in FAs was lower than that in matched normal tissues, though the difference was not statistically significant (Fig. 1e). Similar results were also observed in thyroid cell lines. With the exception of the normal thyroid cell line, Nthy-ori 3–1, all thyroid cancer cell lines evaluated (three PTCs and two anaplastic carcinomas) exhibited negligible levels of SMOC2 expression (Fig. 1f).

### Immunohistochemical analysis for SMOC2 in normal thyroid and various follicular lesions

**Figure 2:**
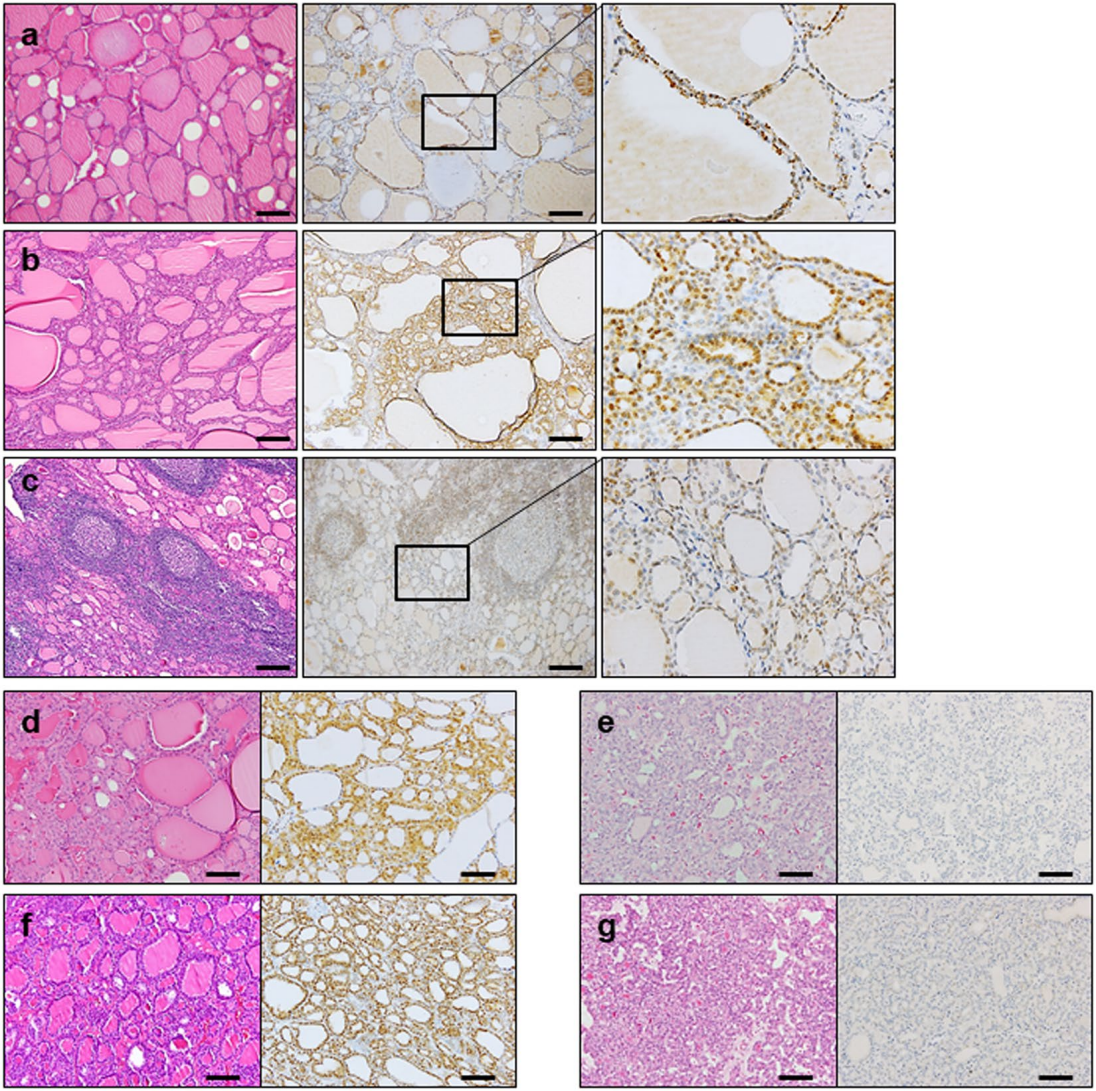
Expression profile of SMOC2 in normal thyroid tissue and various follicular lesions. Immunohistochemistry for SMOC2 was performed on tissue microarrays containing various thyroid lesions including normal thyroid (n = 4) (**a**), nodular hyperplasia (n = 11) (**b**), and lymphocytic thyroiditis (n = 12) cells (**c**). Follicular adenoma (n = 21) with (**d**) or without (**e**) SMOC2 expression. Follicular carcinoma (n = 13) with (**f**) or without (**g**) SMOC2 expression. Scale bars: 50 μm.

To verify the specificity of SMOC2 immunostaining, we performed immunohistochemistry on a human small intestine as SMOC2 was identified as an intestinal stem cell marker to be expressed at the crypt bases^15^. We observed the SMOC2-positive cells confined to the crypt bases, confirming the validity of SMOC2 staining (Fig. S1). We investigated the expression profile of SMOC2 in various human thyroid diseases. As SMOC2 staining showed a diffuse and relatively homogenous expression pattern in most cases, the expression was scored as 0, 1, 2, and 3 according to the stain intensity. The results are summarized in Table 1. Follicular epithelial cells in the normal thyroid expressed SMOC2 with an intensity of 1 or 2 (Fig. 2a). NH showed similar levels of SMOC2 to the normal follicular cells (Fig. 2b), whereas SMOC2 expression was significantly lower in lymphocytic thyroiditis (Fig. 2c). In addition, FAs (Fig. 2d,e) and FCs (Fig. 2f,g) exhibited lower levels of SMOC2 expression than normal thyroid tissues.

**Table 1:** Immunohistochemistry of SMOC2 in thyroid tumors.

	Score (Intensity)	Normal (%)	NH (%)	LT (%)	FA (%)	FC (%)	PTC (%)	MPC (%)
SMOC2	0	0 (0)	0 (0)	1 (8)	3 (14)	6 (46)	127 (38)	60 (49)
	1	1 (25)	3 (27)	10 (84)	11 (52)	4 (31)	138 (41)	53 (43)
	2	3 (75)	7 (64)	1 (8)	6 (29)	2 (15)	52 (15)	10 (8)
	3	0 (0)	1 (9)	0 (0)	1 (5)	1 (8)	21 (6)	0 (0)
Total		4 (100)	11 (100)	12 (100)	21 (100)	13 (100)	338 (100)	123 (100)

NH, nodular hyperplasia; LT, lymphocytic thyroiditis; FA, follicular adenoma; FC, follicular carcinoma; PTC, papillary thyroid carcinoma; MPC, metastatic papillary carcinoma.

### Expression profile of SMOC2 in papillary thyroid carcinoma and lymph node metastasis

**Figure 3:**
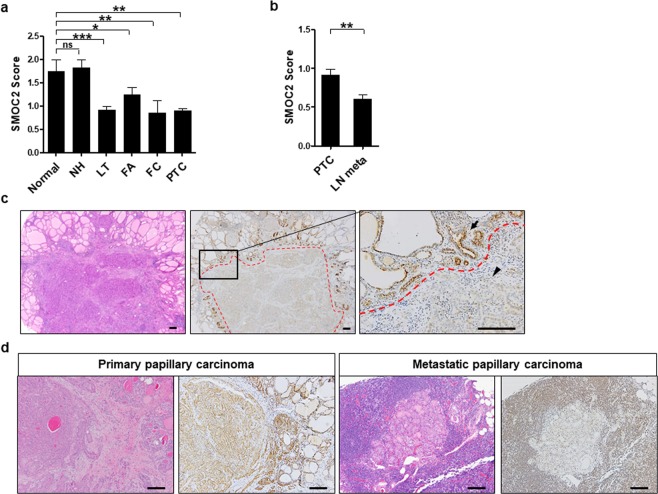
SMOC2 expression in primary and metastatic papillary thyroid carcinomas (PTCs). Immunohistochemistry for SMOC2 was performed on tissue microarrays including PTCs (n = 338) and their metastatic lesions in the lymph nodes (n = 123) (**a**) SMOC2 expression was lower in the PTCs compared to that in normal follicular epithelial cells. (**b**) SMOC2 expression further decreased during lymph node metastasis. (**c**) Representative case of a PTC showing SMOC2 down-regulation (indicated by black arrow head) compared to surrounding normal follicular cells (indicated by black arrow). Red dotted line depicts the boundary between normal thyroid and carcinoma. (**d**) Representative case of a primary PTC showing a dramatic decline in SMOC2 expression upon lymph node metastasis. ^*^P < 0.05, ^**^P < 0.01, ^***^P < 0.001. Scale bar: 100 μm.

Next, we also performed immunohistochemistry for SMOC2 on a large cohort of PTCs (n = 338) including 122 PTCs with lymph node metastasis. The mean level of SMOC2 in the PTCs was substantially lower than that in normal thyroid (*P* = 0.006, Fig. 3a), consistent with the real-time PCR results. For PTCs with lymph node metastasis, we compared the levels of SMOC2 between primary PTCs and metastatic cancers. It was notable that SMOC2 expression further decreased in lymph node metastasis (*P* = 0.002, Fig. 3b). Figure 3c shows a representative PTC case with remarkably decreased SMOC2 expression compared to adjacent normal thyroid follicles and Fig. 3d presents a primary PTC with SMOC2 expression that exhibited no SMOC2 expression upon lymph node metastasis.

### Epigenetic regulation of SMOC2 expression

**Figure 4:**
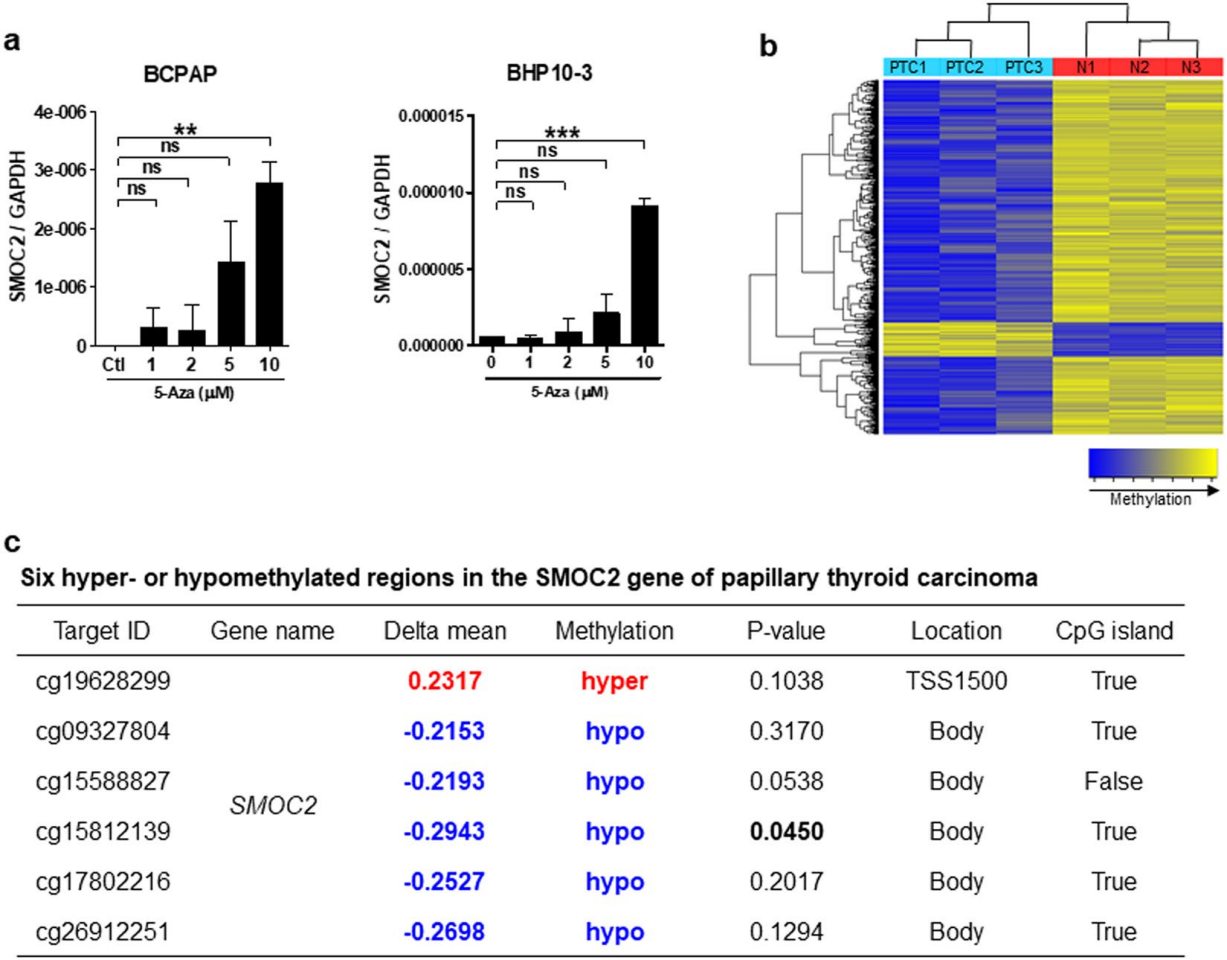
Epigenetic regulation of SMOC2 expression. (**a**) Increased SMOC2 mRNA expression in papillary thyroid carcinoma (PTC) cell lines, BCPAP and BHP10-3, upon 5-Aza-2′-deoxycytidine treatment (ranging from 1 to 10 μM). (**b**) Hierarchical clustering analysis showing the DNA methylation patterns in 3 normal thyroid tissues and 3 primary PTCs. (**c**) Six differentially methylated CpG sites in *SMOC2* gene in PTCs. N, normal; TSS, transcription start site.

To examine whether hypermethylation is involved in the decline of SMOC2 expression in PTC, we treated BCPAP and BHP10-3, two PTC cell lines, with 5-Aza-2′-deoxycytidine (5-Aza), an epigenetic modifier and found a gradual increase of SMOC2 mRNA expression with increasing concentrations (1 to 10 μM) of 5-Aza (Fig. 4a). This result supports an implication of DNA methylation in SMOC2 suppression in PTC. To confirm this finding and identify the differentially methylated loci in *SMOC2* gene, we performed DNA methylation chip analysis with 3 PTCs and adjacent normal tissue samples. Overall, 15540 CpG sites were hypomethylated and 2115 were hypermethylated in PTCs (Figs. 4b and S2). Out of 192 CpG sites localized in the *SMOC2* gene, 6 loci were identified to be differentially methylated in PTCs and only one of them was statistically significant; 5 hypomethylated on the body and one hypermethylated on the transcription start site (Fig. 4c).

### Prognostic value of SMOC2 expression in papillary thyroid carcinomas

**Figure 5:**
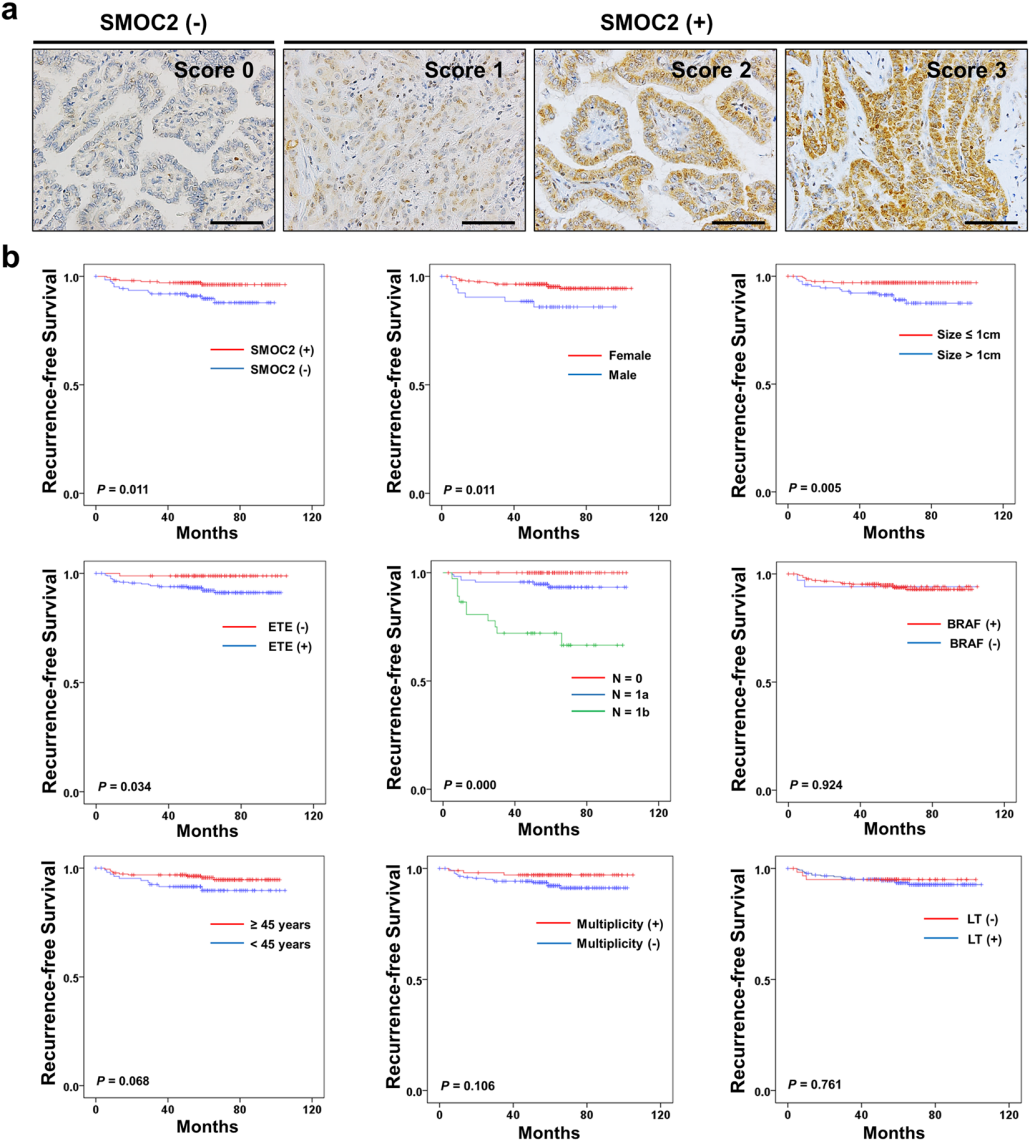
Correlation between SMOC2 expression and recurrence-free survival. (**a**) Representative papillary thyroid carcinoma (PTC) cases of SMOC2 negativity and positivity. (**b**) Recurrence-free survival in PTC patients according to SMOC2 positivity and other clinicopathological features. ETE, extrathyroid extension; LT, lymphocytic thyroiditis.

PTCs with scores of 1, 2, and 3 were considered positive and those with scores of 0 were considered negative (Fig. 5a). In total, 129 cases (38%) of PTC were negative for SMOC2. The clinicopathological relevance of SMOC2 expression is shown in Table 2. SMOC2 positivity was significantly higher in the PTCs with *BRAF* mutation (*P* < 0.001). On the other hand, SMOC2 was not associated with age, gender, tumor size, multifocality, extrathyroid extension, and lymph node metastasis. When evaluating the prognostic significance of SMOC2 expression, SMOC2 positivity was significantly associated with improved recurrence-free survival (*P* = 0.011, Fig. 5b). In addition, female gender (*P* = 0.011), smaller size (*P* = 0.005), absence of extrathyroid extension (*P* = 0.0034), and lower lymph node stage (*P* < 0.001) were found to be associated with better recurrence-free survival. Multivariate analysis, however, revealed that SMOC2 was not statistically significant (*P* = 0.170) and N stage was the only independent prognostic marker (HR = 5.76, *P* < 0.001) (Table 3).

**Table 2:** Association between SMOC2 expression and clinicopathological characteristics.

	Total (%)	SMOC2		*P*-value*
		Negative (%)	Positive (%)	
Patients	338 (100)	129 (38)	209 (62)	
Age				
<45	105 (31)	40 (31)	65 (69)	0.986
≥45	233 (69)	89 (31)	144 (69)	
Sex				
Female	284 (84)	105 (37)	179 (63)	0.300
Male	54 (16)	24 (44)	30 (56)	
Tumor size				
≤1	201 (60)	70 (35)	131 (65)	0.126
>1	137 (40)	59 (43)	78 (57)	
Multifocality**				
Absent	229 (68)	89 (39)	140 (61)	0.701
Present	109 (32)	40 (37)	69 (69)	
Extrathyroid extension				
Absent	82 (24)	33 (40)	49 (60)	0.656
Present	256 (76)	96 (38)	160 (62)	
Lymph node metastasis (n = 277)^#^				
Absent	118 (43)	48 (41)	70 (59)	0.440
Present	159 (57)	56 (35)	103 (65)	
Lymphocytic thyroiditis				
Absent	279 (83)	108 (39)	171 (61)	0.654
Present	59 (17)	21 (36)	38 (64)	
Nodular hyperplasia				
Absent	228 (67)	86 (38)	142 (63)	0.808
Present	110 (33)	43 (39)	67 (61)	
BRAF				
No mutation	34 (10)	24 (71)	10 (29)	<0.001
Mutation	304 (90)	105 (35)	199 (65)	

^*^Pearson chi-square test.

^**^Multifocality includes bilaterality.

^#^277 cases that performed lymph node dissection was included.

**Table 3:** Risk factors for recurrence in all patients with papillary thyroid carcinoma.

	Univariate Analysis		Multivariate Analysis	
	HR (95% CI)	*P*-value	HR (95% CI)	*P*-value
Age (<45 years vs ≥45 years	0.60 (0.24–0.60)	0.278	NA	
Sex (Female vs male)	2.77 (1.04–7.37)	0.042*	1.88 (0.68–5.18)	0.224
Tumor size (≤1 cm vs >1 cm)	3.35 (0.26–8.94)	0.016*	1.45 (0.51–4.11)	0.484
Multifocality (absent vs present)	0.26 (0.06–1.13)	0.073	NA	
Extrathyroid extension (absent vs present)	5.41 (0.72–40.67)	0.101	NA	
N stage (0 vs 1a vs 1b)	7.75 (3.41–17.6)	<0.001*	5.76 (2.42–13.65)	<0.001*
Lymphocytic thyroiditis (absent vs present)	0.78 (0.23–2.73)	0.707	NA	
BRAF mutation (absent vs present)	0.75 (0.17–3.26)	0.700	NA	
SMOC2 (negative vs positive)	0.29 (0.109–0.771)	0.013*	0.49 (0.17–1.36)	0.170

HR, hazard ratio; CI, confidence interval; NA, not applicable; *, statistically significant.

## Discussion

In this study, we thoroughly examined the expression profile of SMOC2 in various thyroid diseases even though some rare thyroid malignancies were not included such as medullary, poorly differentiated, and anaplastic carcinoma. First, we measured the levels of SMOC2 mRNA in a number (n = 47) of paired primary PTC samples using real-time PCR and found that SMOC2 is significantly down-regulated in PTC compared to normal thyroid tissues. This finding was also confirmed in the protein level by the immunohistochemical results for a large number of PTC cases (n = 338). Notably, SMOC2 expression further decreased in the metastatic cancer cells in the lymph nodes compared to primary PTC (Fig. 3b), suggesting a functional implication of SMOC2 down-regulation in the metastatic progression of PTC. Moreover, we discovered that SMOC2 expression is associated with better clinical outcomes for PTC patients although it was not an independent prognostic marker.

According to previous reports on cancer cell lines, the biological functions of SMOC2 are mostly related to pro-oncogenic properties. For example, as mentioned earlier, knocking down SMOC2 in SKBR3 breast cancer cells greatly reduced the ability of the activated Ran mutant to stimulate anchorage-independent growth^11^. Further, Shvab *et al*. demonstrated that SMOC2 overexpression in Ls174T colorectal cancer cells increased motility, proliferation, and metastasis^10^. Additionally, in 482N1 lung adenocarcinoma cells, SMOC2 knockdown suppressed clonal growth and metastatic seeding although it did not affect proliferation or cell death under standard culture conditions^16^. On the other hand, there are a few reports suggesting a tumor suppressive role for SMOC2 in some cancers. In hepatocellular carcinoma cells, SMOC2 expression was shown to significantly reduce cell proliferation, migration, and invasion abilities^12^. Additionally, SMOC2 down-regulation was reported in pancreatic^17^ and breast cancers^18^. These findings indicate that SMOC2 may have different roles depending on the cancer type. Our findings of SMOC2 expression down-regulation in PTC and its association with improved clinical outcomes clearly suggest that SMOC2 likely acts as a tumor suppressor in thyroid cancers.

Interestingly, SMOC2 expression was significantly decreased in lymphocytic thyroiditis, whereas NH showed the same level of SMOC expression as normal thyroid follicles. Since Dailey *et al*. first described the link between Hashimoto’s thyroiditis (HT) and PTC in 1955, there have been conflicting reports regarding the association between HT and PTC despite the fact that a link between cancer and inflammation is well recognized in other organs^19^. Most recently, however, a systemic review of 36 studies published between 1955 and 2016 from 13 countries demonstrated an increased risk of PTC among HT patients^19^. Therefore, our finding of decreased SMOC2 expression in lymphocytic thyroiditis but not in NH, which does not increase the risk of PTC, may suggest that SMOC2 down-regulation in follicular cells in chronic inflammation represents one of the very early molecular changes during the carcinogenesis of PTC.

Genome-wide analysis demonstrated that SMOC2 was hypermethylated in over 70% of pancreatic cancers and less than 10% of normal pancreas^17^, and SMOC2 was one of the candidates for a “highly methylated” signature for endometrial cancers^20^. We also observed that SMOC2 expression increased after 5-Aza treatment in the PTC cell lines. Therefore, it is reasonable to hypothesize that SMOC2 down-regulation in PTC is the result of epigenetic modification. To identify the differentially methylated CpG sites in *SMOC2* gene, we performed DNA methylation chip analysis with 3 primary PTCs and corresponding normal thyroid tissues. Overall, 88% of differentially methylated genes were hypomethylated and 12% were hypermethylated in PTCs, consistent with the previous study reporting that PTCs had a higher proportion of hypomethylated probes^21^. Top 10 hyper- or hypomethylated loci in PTCs are summarized in Supplementary Table S1. When looking into the 192 CpG sites in *SMOC2* gene, it was unexpected to find that only 6 were differentially methylated; 5 hypomethylated loci in the gene body and one hypermethylated site in the transcription start site (TSS). Although hypomethylation is predominant in *SMOC2* gene, it is possible that the hypermethylation in the TSS may be contributing to SMOC2 down-regulation in PTC since DNA methylation in the promoter region is the major determinant of gene suppression^22^. However, because the number of PTC samples included in the methylation analysis was very small and the hypermethylated CpG site did not reach statistical significance, it needs to be confirmed by pyrosequencing.

*BRAF* V600E is the most common oncogenic mutation that aberrantly activates the MAP kinase pathway in PTC and has mostly been demonstrated to be associated with aggressive clinical outcomes^23^. However, the prognostic significance of *BRAF* mutation is controversial in Korea where the prevalence of *BRAF* mutation is much higher than in Western countries^24^. Indeed, we found that 90% of PTC had a *BRAF* mutation, which was not associated with recurrence-free survival. Remarkably, SMOC2 positivity was higher in PTC with *BRAF* mutation (Table 2). Although we used immunohistochemistry to detect the presence of *BRAF* (V600E) mutation instead of using direct sequencing or mutant-specific PCR, the mutation-specific antibody VE1 has been demonstrated to be a reliable and highly specific antibody for *BRAF* mutation in PTC^25,26^. To examine whether *BRAF* mutation is involved in the regulation of SMOC2 expression, we transfected the normal thyroid cell line Nthy-ori 3-1 with the *BRAF* mutant and found a slight increase in SMOC2 expression (Fig. S3). However, this was not consistent with our finding that a majority of PTCs had *BRAF* mutation and expressed lower levels of SMOC2 than the normal thyroid. Therefore, it is unlikely that *BRAF* mutation is one of the major determinants of SMOC2 expression.

In summary, SMOC2 is normally expressed in thyroid follicular epithelial cells and the expression was the same in NH. SMOC2 expression was significantly lower in lymphocytic thyroiditis and in follicular tumors including FAs and carcinomas. In particular, the complete loss of SMOC2 observed in 38% of PTC cases was associated with the absence of *BRAF* mutation and worse clinical outcomes. These findings suggest that SMOC2 can be a biomarker to predict the prognosis of PTC patients.

## Materials and Methods

### Subjects

Papillary thyroid carcinomas (PTCs) were collected from 338 patients who underwent surgical resection at Jeju National University Hospital, Jeju, Korea from 2008 to 2012. Clinicopathological data, including the patient age, gender, tumor size, histological type, multiplicity, presence of extrathyroid extension, and N stage (AJCC 7^th^ edition), were obtained by reviewing the pathological reports. Patient outcomes included information on the recurrence and follow-up time. Normal thyroid (n = 4), nodular hyperplasia (NH) (n = 11), lymphoid thyroiditis (LT) (n = 12), follicular adenoma (FA) (n = 21), follicular carcinoma (FC) (n = 13) samples were obtained from total thyroidectomy or lobectomy specimen performed at Jeju National University hospital. In addition, 47 paired fresh-frozen PTC tissues with matched non-cancerous tissues, and 19 paired FAs and non-tumor tissues were provided by the Jeju National University Hospital Biobank, a member of the National Biobank of Korea. This study was approved by the Institutional Review Board (IRB) of Jeju National University Hospital (IRB No. 2016-08-006) and informed consent was waved due to the retrospective nature of this study. All experiments were approved by IRB and performed in accordance with relevant guidelines and regulations.

### Tissue microarray construction

Twenty two tissue microarrays (TMAs) including 338 primary PTCs, 123 metastatic PTCs in the lymph nodes, 4 normal thyroid, 11 NH, 12 LT, 21 FA, and 21 FC cases were generated. In brief, the representative tumor portion containing more than 70% of cell population was marked in each case through microscopic examination. Tumor core of 4- mm diameter was obtained from the matched area of formalin-fixed, paraffin-embedded tissue block and arranged in a new recipient paraffin block using a trephine apparatus (SuperBioChips Laboratories, Seoul, Korea).

### Immunohistochemistry

According to the manufacturer’s instructions, immunohistochemistry was performed on 4-μm TMA sections using a Ventana BenchMark XT Staining systems (Leica Microsystems, Wetzlar, Germany). The primary antibodies used were anti-SMOC2 (OriGene, Rockville, MD; 1:30) and anti-BRAF (V600E) (Spring Bioscience, VE1; 1:50). Cytoplasmic SMOC2 staining was scored from 0 to 3 according to the stain intensity and each case was considered as positive when score is more than 1.

### RNA extraction and Quantitative real-time PCR

Total RNA was extracted from fresh-frozen 47 PTCs, 19 Fas and their corresponding normal thyroid tissues using TRIZOL reagent (Invitrogen, Carlsbad, CA, USA). RNA (1–2 μg) was reverse-transcribed with oligo-dT primers and the GoScript reverse transcription system (Promega, Madison, Wisconsin). Complementary DNA (cDNA) was subsequently used to perform real-time PCR with Premix EX Taq (Takara bio, Shiga, Japan) according to the manufacturer’s recommendations, and the cycling conditions were as follows: initial denaturation for 30 s at 95 °C, which was followed by 40 cycles of 95 °C for 1 s and 60 °C for 20 s in a StepOne Plus real-time PCR system (Applied Biosystems, Foster City, CA). The TaqMan gene expression assays were used as follows: Hs01591663_m1 (SMOC2) and Hs0275899_g1 (GAPDH). GAPDH served as the endogenous control.

### Cell lines and reagents

Six human thyroid cell lines including a normal thyroid cell line (Nthy-ori 3-1), three PTC cell lines (BCPAP, BHP, SNU790), and two anaplastic carcinoma cell lines (SNU80, 8505 C) were generously provided by Dr. Jae Kyung Myung (Korea Cancer Center Hospital). Cell lines were cultured in RPMI1640 media containing 10% fetal bovine serum and antibiotics (penicillin G and streptomycin) in a humidified incubator containing 5% CO2. 5-Aza was purchased from Sigma Aldrich.

### Transfection of BRAF

Full-length cDNA encoding wild type *BRAF* (pCMV-BRAF) and mutant *BRAF* (pCMV-BRAF(V600E)) were purchased from OriGene (Rockville, MD) Cells were seeded at 1 × 10^6^ cells per well in a six-well plate and transfected with 2.5 μg of pCMV-BRAF, or pCMV-BRAF(V600E) using Lipofectamine 3000 transfection reagent (Invitrogen, Carlsbad, CA). One day after transfection, cells were subjected to real-time PCR.

### DNA methylation chip analysis

Genome-wide DNA methylation profiling for 3 primary papillary carcinomas and matched normal thyroid tissues was performed at Macrogen (Seoul, South Korea) using the Illumina Infinium MethylationEPIC BeadChip kits (Illumina, San Diego, CA). Genomic DNA was bisulfite-converted using the EZ DNA methylation kit (Zymo Research, Orange, CA) according to the manufacturer’s protocols. Raw data were extracted as beta values for each CpG for each sample using R watermelon package. Beta values were calculated by subtracting background using negative controls on the array and taking the ratio of the methylated signal intensity against the sum of both methylated and unmethylated signals. A beta value of 1–1.0 was reported significant percent methylation, from 0% to 100%, respectively, for each CpG site. Array CpG probes that have detection p-value ≥ 0.05 (similar to signal to noise) in over 25% samples were filtered out. (We applied a filtering criterion for data analysis; good signal value was required to obtain a detection p-value < 0.05). And then filtered data was background correction & dye bias equalization by R methylumi & lumi package. To reduce Infinium I and Infinium II assay bias, corrected signal value was normalized by BMIQ (Beta Mixture Quantile) method. Differentially expressed methylation list were determined using |delta_mean | ≥0.2 (the difference of methylation signal, avg beta of Case – avg beta of Control) and p-value < 0.05 of independent t-test in which the null hypothesis was that no difference exists among 2 groups. All data analysis and visualization of differentially expressed genes was conducted using R 3.3.3 (www.r-project.org).

### Statistical analysis

Statistical analyses were performed with the PASW 18.0 statistical software program (IBM SPSS Statistics, Chicago, IL) and Prism version 5.0 (GraphPad Software, Inc., San Diego, CA). Between-group comparisons of the real-time PCR data were analyzed using Student’s t-test. The correlation between SMOC2 positivity and clinico-pathological characteristics were tested using Pearson’s chi-square test. Recurrence-free survival curves were estimated using the Kaplan-Meier method, and the log-rank test was used to compare groups. The Cox proportional hazards model was used for comparing hazard ratios in multivariate analyses. A *P*-value < 0.05 was considered statistically significant.

## Supplementary information

supplementary information.
