# Supplementary material for: Downregulation of SMOC2 expression in papillary thyroid carcinoma and its prognostic significance
Source: Sci Rep. 2020 Mar 17;10:4853. doi: 10.1038/s41598-020-61828-z (PMC7078233; doi:10.1038/s41598-020-61828-z)
Supplement: Supplementary file 1 — supplementary information. [file 41598_2020_61828_MOESM1_ESM.pdf]

Downregulation of SMOC2 expression in papillary thyroid carcinoma and its prognostic significance

(Running title: SMOC2 expression in thyroid tumors)

Hye Sung Kim[1], Jae Hyuck Choi[2], Jae Young Lee[1], JiHoon Kang[3], Jae Kyung Myung[3,4], Woo Ho Kim[5], Bo Gun Jang[1]

[1]Department of Pathology, Jeju National University School of Medicine and Jeju National University Hospital, Jeju, South Korea

[2]Department of Surgery, Jeju National University School of Medicine and Jeju National University Hospital, Jeju, South Korea

[3]Laboratory of Radiation Exposure & Therapeutics, National Radiation Emergency Medical Center, South Korea Institute of Radiological & Medical Sciences, Seoul, South Korea

[4]Department of Pathology, Korea Cancer Center Hospital, Seoul, South Korea

[5]Department of Pathology, Seoul National University College of Medicine, Seoul, South Korea

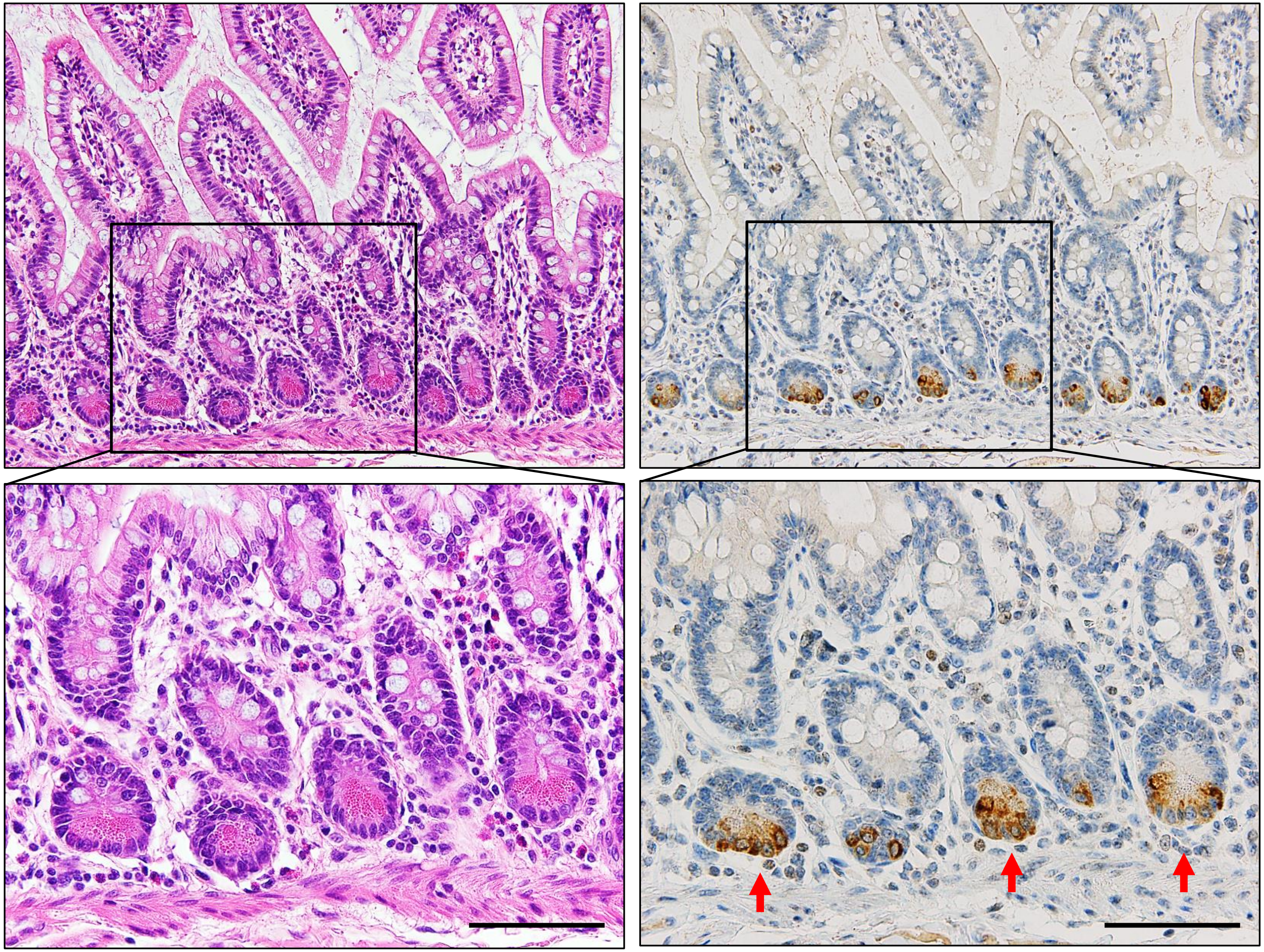

Fig. S1. Validation of an antibody for SMOC2 expression in human tissue. Immunohistochemistry for SMOC2 demonstrated the specific marking of SMOC2-positive stem cells located in the human small intestine (indicated by red arrows). Scale bar : 50 μm.

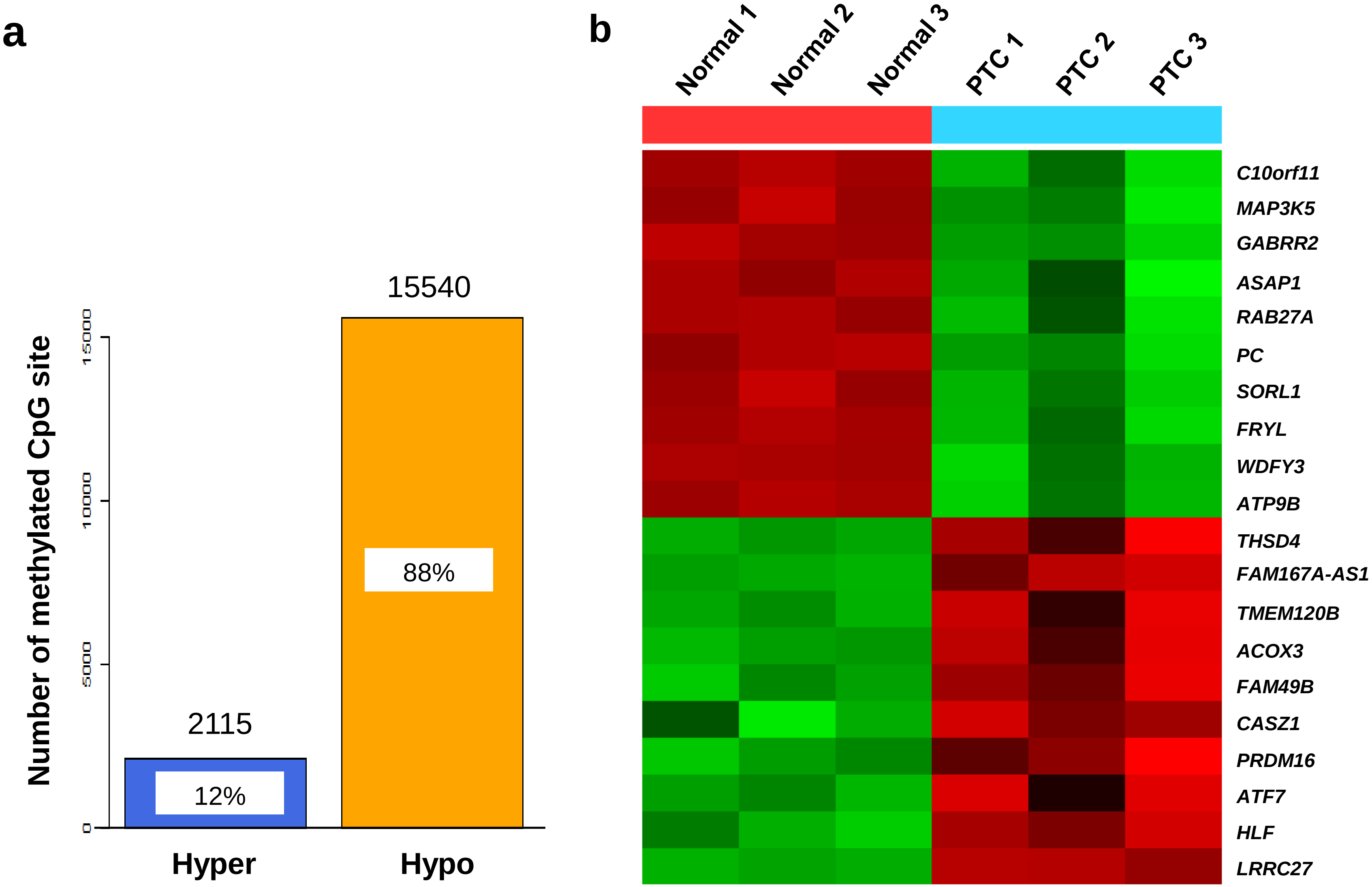

Fig. S2. (**a**) Number of differentially methylated CpG sites in the papillary thyroid carcinomas (PTCs). (**b**) Heatmap of top 10 hypo- or hypermethylated loci in 3 PTCs and corresponding normal thyroid tissues. Hyper, hypermethylation; Hypo, hypomethylation.

Table S1 TOP 10 hyper- or hypomethylated probes for papillary thyroid carcinomas (PTCs)

Target ID	Gene name	Normal Mean	PTC Mean	PTC-Normal.delta_mean	Methylation	P-value	Chr.
cg11127535	*THSD4*	0.351929292	0.854332372	**0.50240308**	**hyper**	**0.022441966**	15
cg12516759	*FAM167A-AS1*	0.225811253	0.732823509	**0.507012255**	**hyper**	**0.005840797**	8
cg26531879	*TMEM120B*	0.254770196	0.764437704	**0.509667508**	**hyper**	**0.025283343**	12
cg17781866	*ACOX3*	0.408311875	0.923477273	**0.515165398**	**hyper**	**0.016108876**	4
cg16871855	*FAM49B*	0.403088913	0.919936788	**0.516847874**	**hyper**	**0.003889197**	8
cg18236877	*CASZ1*	0.249612927	0.785854576	**0.536241649**	**hyper**	**0.005516102**	1
cg02404410	*PRDM16*	0.235923002	0.783189139	**0.547266137**	**hyper**	**0.012183483**	1
cg27549963	*ATF7*	0.191460286	0.769227543	**0.577767258**	**hyper**	**0.033125128**	12
cg24297504	*HLF*	0.158597286	0.782584144	**0.623986859**	**hyper**	**0.000601355**	17
cg14683065	*LRRC27*	0.025913281	0.768267087	**0.742353806**	**hyper**	**0.000290316**	10
cg17458910	*C10orf11*	0.897486691	0.132637114	**-0.764849577**	**hypo**	**0.006929317**	10
cg07474842	*MAP3K5*	0.841035594	0.120535305	**-0.720500289**	**hypo**	**0.003535914**	6
cg03301058	*GABRR2*	0.829753412	0.118956176	**-0.710797236**	**hypo**	**0.000666548**	6
cg11275536	*ASAP1*	0.783112562	0.074865302	**-0.70824726**	**hypo**	**0.018265104**	8
cg02392359	*RAB27A*	0.897728445	0.190549418	**-0.707179027**	**hypo**	**0.013661737**	15
cg15073665	*PC*	0.854957318	0.149991606	**-0.704965712**	**hypo**	**0.00151702**	11
cg13606889	*SORL1*	0.833858412	0.13029328	**-0.703565132**	**hypo**	**0.001044148**	11
cg14036584	*FRYL*	0.897026482	0.205228173	**-0.691798309**	**hypo**	**0.00814241**	4
cg16011130	*WDFY3*	0.829876605	0.138618485	**-0.691258119**	**hypo**	**0.007103676**	4
cg09304470	*ATP9B*	0.904643977	0.213443613	**-0.691200364**	**hypo**	**0.004023832**	18

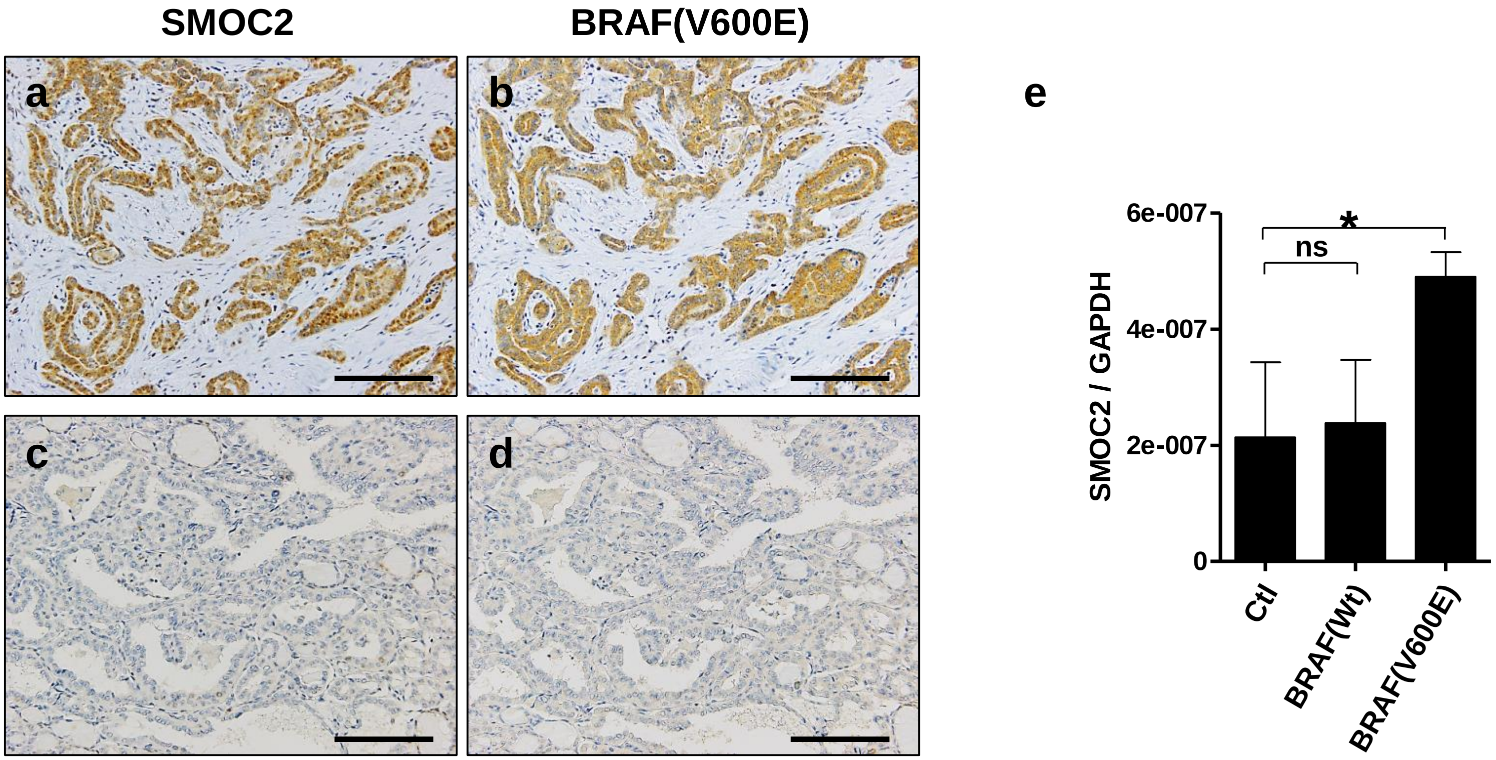

Fig. S3. Correlation of SMOC2 with BRAF mutation. Representative case of a PTC with SMOC2 and BRAF(V600E) expression (**a** and **b**) and a PTC without SMOC2 and BRAF(V600E) expression (**c** and **d**) (**e**) Increased SMOC2 expression upon mutant BRAF transfection in N-thy1 cell line. Wt, Wild type; Scale bar : 100 μm.
